# Supplementary material for: Phytochemicals from Purwoceng (Pimpinella pruatjan) and Their Potential in Chronic Disease Prevention: Focus on Kidney Health
Source: Int J Mol Sci. 2025 Aug 29;26(17):8404. doi: 10.3390/ijms26178404 (PMC12428217; doi:10.3390/ijms26178404)
Supplement: Supplementary file 1 [file ijms-26-08404-s001.zip › ijms-3781832-supplementary.pdf]

Supplementary materials

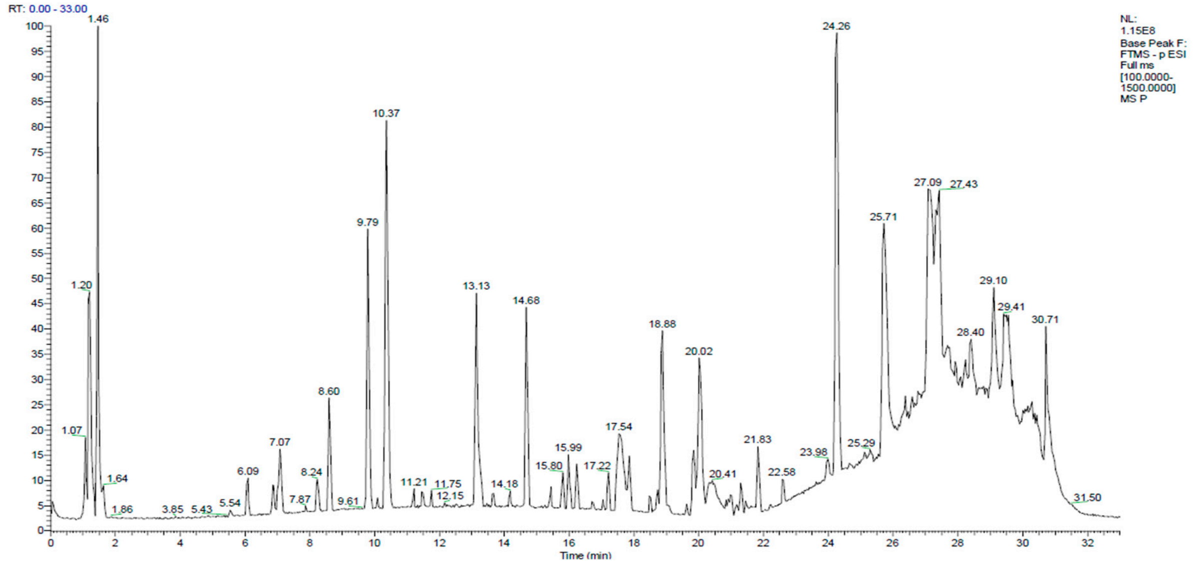

Figure S1. LC-HRMS analysis. Basepeak chromatogram (BPC) of Purwoceng root ethanolic extract in negative mode following analysis by UHPLC-Q -Orbitrap - HRMS

Table S1. Putative compounds detected in the purwoceng ethanolic root extract by LC-HRMS.

No	Name	Formula	LogP	Annot. Delta Mass [ppm]	Retention Time [min]	% area	Group of compounds
1	Hexitol	$C_6 H_{14} O_6$	-3.59	-3.68	1.077	0.81	carbohydrate
2	Unknown	$C_5 H_7 N O_3$	-	-7.51	1.202	2.41	-
3	Unknown	-	-	-	1.458	2.57	-
4	Unknown	$C_7 H_7 N O_3$	-	-5.13	6.079	0.55	-
5	Unknown	$C_7 H_6 O_2$	-	-7.63	7.077	0.96	-
5	Suberic acid	$C_8 H_{14} O_4$	1.50	-3.81	8.267	0.35	Dicarboxylic acid
7	(E)-Ferulic acid	$C_{10} H_{10} O_4$	1.50	-3.24	8.594	1.26	Hydroxycinnamic acid
8	3,4-dihydroxyphenylacetic acid	$C_8 H_8 O_4$	0.72	-4.96	10.356	4.70	Dopamine derivative
9	Sebacic acid	$C_{10} H_{18} O_4$	2.28	-2.48	11.195	0.40	Fatty acid
10	(-)-pinellic acid	$C_{18} H_{34} O_5$	3.02	-0.31	13.138	3.32	Fatty acid
11	Unknown	$C_{14} H_{14} O_3$	-	-	14.677	2.37	-

12	Unknown	$C_{18}H_{28}O_4$	-	-	17.525	0.59	-
13	(10E,12Z)-9-Hydroperoxy-10,12-octadecadienoic acid	$C_{18}H_{32}O_4$	5.35	0.08	17.875	0.38	Unsaturated Fatty acid
14	Unknown	$C_{27}H_{52}NO_9P$	-	-	18.865	3.42	-
15	13S-hydroxyoctadecadienoic acid	$C_{18}H_{32}O_3$	4.90	-0.9	20.039	2.97	Unsaturated fatty acid
16	Ricinoleic Acid	$C_{18}H_{34}O_3$	5.08	-0.37	20.823	0.67	Unsaturated Fatty acid
17	(9Z,11E)-13-Oxo-9,11-octadecadienoic acid	$C_{18}H_{30}O_3$	5.06	-0.10	21.309	0.67	Unsaturated Fatty acid
18	Unknown	$C_{20}H_{26}O_4$	-	-	21.823	1.10	-
19	13S-hydroxyoctadecadienoic acid	$C_{18}H_{32}O_3$	4.90	-0.90	22.215	0.38	Unsaturated Fatty acid
20	Juniperic acid	$C_{16}H_{32}O_3$	4.52	-0.54	24.248	7.28	Fatty acid
21	Linoleic Acid	$C_{18}H_{32}O_2$	5.88	-0.81	25.722	1.99	Unsaturated Fatty acid
22	Palmitic Acid	$C_{16}H_{32}O_2$	5.55	-1.00	27.111	0.38	Fatty acid
23	Oleic acid	$C_{18}H_{34}O_2$	6.10	-0.57	27.376	0.32	Unsaturated Fatty acid
